# Association of cardiometabolic multimorbidity with risk of late-life depression: a nationwide twin study

**DOI:** 10.1192/j.eurpsy.2024.1775

**Published:** 2024-09-25

**Authors:** Wenzhe Yang, Weiwei Li, Shuqi Wang, Xiuying Qi, Zhuoyu Sun, Abigail Dove, Weili Xu

**Affiliations:** 1 Department of Epidemiology and Biostatistics, School of Public Health, Tianjin Medical University, Tianjin, China; 2 Tianjin Key Laboratory of Environment, Nutrition and Public Health, Tianjin, China; 3 Center for International Collaborative Research on Environment, Nutrition and Public Health, Tianjin, China; 4 Aging Research Center, Department of Neurobiology, Care Sciences and Society, Karolinska Institutet, Stockholm, Sweden

**Keywords:** cardiometabolic disease, depression, Swedish Twin Registry, twin study

## Abstract

**Background:**

Cardiometabolic diseases (CMDs) including heart disease, stroke, and type 2 diabetes have been individually linked to depression. However, their combined impact on depression risk is unclear. We aimed to examine the association between cardiometabolic multimorbidity and depression and explore the role of genetic background in this association.

**Methods:**

Within the Swedish Twin Registry, 40,080 depression-free individuals (mean age 60 years) were followed for 18 years. Cardiometabolic multimorbidity was defined as having ≥2 CMDs. CMDs and depression were ascertained based on the National Patient Register. Cox regression was used to estimate the CMD-depression association in a classical cohort study design and a matched co-twin design involving 176 twin pairs. By comparing the associations between monozygotic and dizygotic co-twins, the contribution of genetic background was estimated.

**Results:**

At baseline, 4809 (12.0%) participants had one CMD and 969 (2.4%) had ≥2 CMDs. Over the follow-up period, 1361 participants developed depression. In the classical cohort design, the multi-adjusted hazard ratios (95% confidence interval [CIs]) of depression were 1.52 (1.31–1.76) for those with one CMD and 1.83 (1.29–2.58) for those with ≥2 CMDs. CMDs had a greater risk effect on depression if they developed in mid-life (<60 years) as opposed to late life (≥60 years). In matched co-twin analysis, the CMD-depression association was significant among dizygotic twins (HR = 1.63, 95% CI, 1.02–2.59) but not monozygotic twins (HR = 0.90, 95% CI, 0.32–2.51).

**Conclusions:**

Cardiometabolic multimorbidity is associated with an elevated risk of depression. Genetic factors may contribute to the association between CMDs and depression.

## Introduction

Depression is a global public health issue that affects approximately 350 million people worldwide [1] and is estimated to become the leading cause of disease burden globally by 2030 [2, 3]. Distinct from regular mood changes and feelings about daily life, depression brings profound suffering to individuals, impairs interpersonal relationships and social functioning, and is associated with disability [1]. Although depression occurs more commonly in earlier life (i.e., 20–40 years), there is a second peak of prevalence in later life (i.e., 50–70 years) [4]. Early-life depression is mainly influenced by family history of depression and stress events, while late-life depression is related to vascular dysfunction and somatic disease [5, 6]. Furthermore, late-life depression has been linked to much higher risk of mortality compared with early-life depression [7]. Despite this, few studies have addressed the relationship between somatic multimorbidity and depression in late life.

Cardiometabolic diseases (CMDs), a cluster of diseases including type 2 diabetes (hereafter diabetes), heart disease, and stroke [8–11], increase in prevalence during older age. Previous studies have shown that diabetes [12, 13], heart disease [14, 15], and stroke [14, 16] are individually related to depression risk. As the population ages and people live longer with CMDs, cardiometabolic multimorbidity – i.e., the coexistence of two or more CMDs – has become increasingly common among older adults [8]. A previous study based on UK Biobank data reported a dose-dependent association between greater number of co-morbid CMDs and depression, although the cross-sectional study design leaves the temporality of the association unclear [17]. However, limited studies have examined the longitudinal association between cardiometabolic multimorbidity and the risk of incident depression in late life. In addition, given the variability of CMDs as chronic disorders with a potentially decades-long time course, it is unclear whether depression risk differs depending on the age of occurrence of CMDs.

Women have a higher risk of depression than men due to hormone differences and psychosocial factors [4]. Thus, it is plausible that a sex difference might exist in the CMD-depression association. Moreover, accumulating evidence shows that genetic and early-life environmental factors (such as fetal environment, upbringing environment, and family socioeconomic status) might influence the development of both CMDs and depression [4, 18–20]. A twin study is particularly useful to assess whether and to what extent these unmeasured familial factors play a role in the CMDs-depression association, as twins represent naturally matched pairs between whom the potential confounding influence of genetic background and early-life environment can be controlled [21].

In this prospective cohort study of over 40,000 twins, we aimed to (1) examine the association between cardiometabolic multimorbidity and the risk of late-life depression, (2) investigate whether this association might differ between men and women, and (3) explore the extent to which the CMD-depression association can be explained by genetic and early-life environmental factors.

## Methods

### Study population

**Figure 1.**
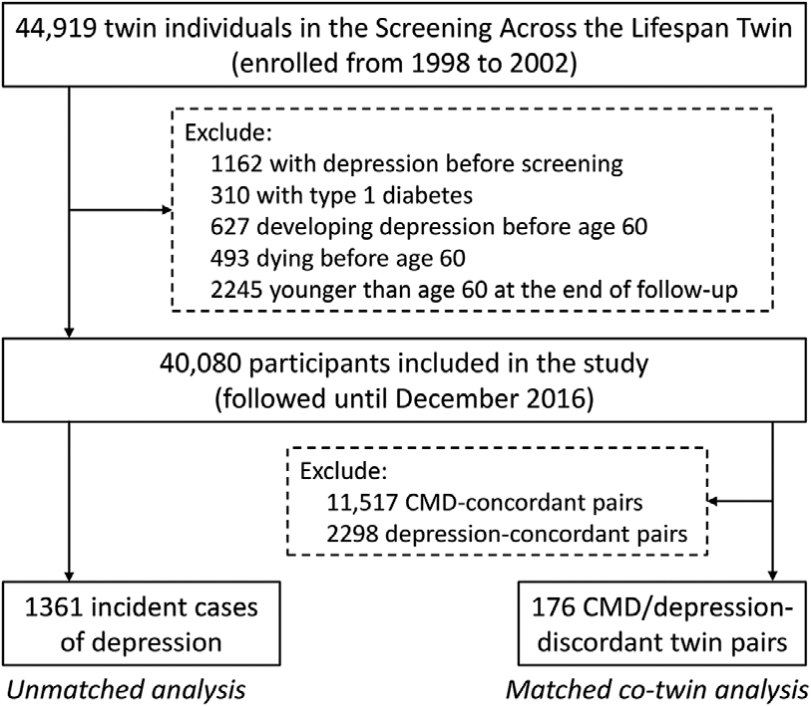
Flowchart of the study population.

Study participants were drawn from the Swedish Twin Registry (STR), which includes all living twins born in Sweden since the late 1800s [21]. Between 1998 and 2002, a total of 44,919 twin individuals aged ≥40 years participated in the Screening Across the Lifespan Twin (SALT) survey (a computer-assisted telephone interview) and were followed via medical records from the Swedish National Patient Register (NPR) for up to 18 years (until 2016). Of all participants, we excluded 310 participants with prevalent type 1 diabetes, 1162 who had depression before screening, 627 who developed depression before age 60, 493 who died before age 60, and 2245 who were younger than 60 at the end of follow-up (December 31, 2016). A total of 40,080 participants were ultimately included in the current study (Figure 1).

All participants provided informed consent, and this study was approved by the Regional Ethics Board at Karolinska Institutet (Dnr: 97: 051), Stockholm, Sweden.

### Data collection

Information on participants’ zygosity status, socio-demographic characteristics (age, sex, educational level, and marital status), lifestyle behaviors (smoking history, alcohol consumption, and physical activity), height, weight, and medical history was collected in the SALT baseline survey. In addition, the onsets of diseases were identified based on the use of medications according to the Swedish Prescribed Drug Register and/or International Classification of Disease (ICD) codes in the NPR. The NPR was established in the 1960s, and complete coverage of somatic and psychiatric inpatient diagnoses was achieved in 1987. Since 2001, the NPR has also included diagnoses from specialized outpatient clinics, with >87% coverage [22]. Currently, more than 99% of all inpatient and outpatient diagnoses are registered in the NPR [23].

Zygosity status was grouped as monozygotic (MZ), dizygotic (DZ), and undetermined zygosity. Zygosity status was identified based on the participants’ answers to the question: “During childhood, were you and your twin partner as like as “two peas in a pod” or not more alike than siblings in general?” If both co-twins answered that they were “as alike as two peas in a pod,” they were classified as MZ; if both co-twins answered that they were no more alike than siblings, they were classified as DZ; and if the co-twins gave inconsistent answers or only co-twin of the pair answered the question, zygosity was classified as undetermined [21]. This method for distinguishing MZ and DZ twins has been validated and shown to be highly accurate (nearly 99%) [21].

Educational level was dichotomized as <8 years versus ≥8 years based on the number of years of formal education. Marital status was categorized as married/cohabitating versus single (including divorced, widowed, and living alone). Smoking status was categorized as non-smokers versus former/current smokers. Alcohol consumption was dichotomized as no/mild drinking versus heavy drinking. Physical activity level was assessed through a survey question on annual exercise patterns and dichotomized as low (“almost never,” “much less than average,” and “less than average”) versus high (“average,” “more than average,” “much more than average,” and “maximum”). Height and weight were collected based on self-report, and body mass index (BMI) was calculated as weight in kilograms (kg) divided by height in meters squared (m^2^). Baseline hypertension was ascertained through the linkage to the NPR.

### Assessment of cardiometabolic diseases

CMDs were defined as diabetes, heart disease (including coronary heart disease, atrioventricular block, cardiac arrhythmias, and heart failure), and stroke (including ischemic, hemorrhagic, and unspecified stroke), following previous studies examining the impact of CMDs on health outcomes [8, 9, 24]. CMDs were ascertained at baseline using multiple data sources. Diabetes was identified according to the self-reported medical history, use of glucose-lowering drugs, and NPR (ICD-7, 260; ICD-8, 250; ICD-9, 250.00, 250.02, 250.10, 250.12, 250.20, 250.22, 250.30, 250.32, 250.40, 250.42, 250.50, 250.52, 250.60, 250.62, 250.70, 250.72, 250.80, 250.82, 250.90, 250.92; and ICD-10, E11–E14). Heart disease and stroke were identified based on diagnosis information extracted from the NPR. The ICD codes used for diagnoses of heart disease included: ICD-7, 420; ICD-8, 410–414; ICD-9, 410–414, 426–428; and ICD-10: I20–I25, I44, I45, I48–I50. The ICD codes used for diagnoses of stroke included: ICD-7, 330–334; ICD-8: 430–434, 436–438; ICD-9: 430–434, 436, 437; and ICD-10: I60–I63, I67, I68.

Participants were dichotomized as CMD-free or having any CMD. We also grouped participants according to their total number of CMDs: single CMD (i.e., diabetes, heart disease, or stroke alone) and cardiometabolic multimorbidity (two or more comorbid CMDs). In particular, different CMD patterns may affect depression differently. Therefore, to explore the effects of specific constellations of comorbid CMDs on depression, cardiometabolic multimorbidity was further categorized as diabetes/heart disease, diabetes/stroke, heart disease/stroke, and diabetes/heart disease/stroke in exploratory analyses. Age of CMD occurrence was ascertained according to the earliest recorded date of diabetes, heart disease, or stroke diagnosis in the NPR and/or glucose-lowering medication usage in the Prescribed Drug Register (only for diabetes). The age at which participants’ first and second (in the case of cardiometabolic multimorbidity) CMD occurred was dichotomized as mid-life (<60 years) or late life (≥60 years).

### Ascertainment of depression

Depression was ascertained based on the diagnoses of depression from the NPR. The following ICD codes were used to exclude prevalent depression and identify incident depression: ICD-7, 314.99; ICD-8, 296.00, 298.00, 300.40, 300.41, 790.20; ICD-9, 296C, 296D, 296 W, 298A, 300E, 309A, 309B, 311X; and ICD-10, F32, F33, F34.1, F41.2.

### Statistical analysis

Baseline characteristics of participants by CMD status were assessed using Chi-square (*χ* ^2^) tests for categorical variables and one-way analysis of variance for continuous variables. Statistical analyses were then conducted in two major steps: (i) a classical cohort study design including all twin individuals (i.e., unmatched analysis) and (ii) a matched co-twin analysis involving twin pairs discordant for both CMDs and depression.

#### Classical cohort study design

In the classical cohort study design, Cox regression models were used to estimate the hazard ratio (HR) and 95% confidence interval (CI) of depression risk in participants with a single CMD and with cardiometabolic multimorbidity (reference: CMD-free). Follow-up time (in years) was calculated as the time from baseline to depression diagnosis, death, or the end of follow-up (31 December 2016), whichever occurred first. The model additionally included a sandwich estimator to account for the clustering of co-twins within a pair. Furthermore, to determine whether the timing of CMD occurrence affects depression risk, we reran the Cox models after stratifying participants with CMDs by the age of CMD onset (mid-life [<60 years] versus late-life [≥60 years]).

To assess possible sex differences in CMD-depression association, models were repeated after stratifying by sex. We tested the multiplicative interaction between sex and CMD status by entering their cross-product term (sex × CMDs) into the model. We also evaluated the combined effect of CMDs and sex on depression risk by adding a 4-category indicator variable combining CMD status (yes versus no) and sex (male versus female) into the model. The additive interaction between CMD status and sex was evaluated using relative excess risk of interaction (RERI) and the attributable proportion of interaction (API).

#### Matched co-twin analysis

The aim of the matched co-twin analysis was to assess the roles of genetic and early-life environmental factors in the CMDs-depression association and to further control for the clustering of twin pairs. The analysis was restricted to 176 twin pairs who were discordant for both CMD status and depression status (i.e., each twin pair contained one individual with CMDs and one individual without CMDs, only one of whom developed depression or both of whom developed depression at different times). Cox regression models were used to estimate the association between CMDs and depression in DZ twin pairs (*n =* 148) and MZ twin pairs (*n =* 28) separately. The confounding influence of genetic background can be fully controlled among MZ twin pairs, who share 100% of their genetic background (compared to only 50% for DZ twin pairs). Therefore, if the association observed in the classical cohort study design is attenuated in MZ pairs compared with DZ pairs, this would suggest that genetic factors common to both CMDs and depression contribute to the association [14].

#### Confounder adjustment and sensitivity analyses

Analyses were first adjusted for age, sex, and education, and then further adjusted for marital status, smoking status, alcohol consumption, physical activity level, BMI, and hypertension. Notably, age was not included as a covariate in the co-twin analyses since age is always the same for both members of a twin pair. Similarly, in analyses of MZ twins, sex was also not included since such twin pairs are always same-sex. Missing values for education (*n =* 1461), marital status (*n =* 939), smoking status (*n =* 1411), alcohol consumption (*n =* 1505), physical activity (*n =* 3544), and BMI (*n =* 2199) were imputed using fully conditional specification with 5 imputations.

The following sensitivity analyses were also performed: (1) excluding 6918 participants with missing values for covariates and (2) excluding 64 participants who developed depression in the first 2 years of follow-up to minimize reverse causation, and (3) estimating the associations of each individual CMD (diabetes, heart disease, and stroke) with late-life depression separately.

Analyses were performed using SAS 9.4 (SAS Institute), with statistical significance defined as *P* < 0.05 (two-tailed).

## Results

### Characteristics of the study population

The sample was composed of 40,080 twin individuals (mean age 60.47 ± 10.69 years), including 18,715 (46.7%) men and 21,365 (53.3%) women. Of all participants, 5778 (14.4%) had at least one CMD, including 4809 (12.0%) with a single CMD and 969 (2.4%) with cardiometabolic multimorbidity. Compared to CMD-free individuals, those with cardiometabolic multimorbidity were more likely to be older, male, single, less physically active, and have a lower education level, higher BMI, and higher prevalence of hypertension. No statistically significant difference in smoking status or alcohol consumption was observed between the three groups (Table 1).

**Table 1.** Baseline characteristics of the study population by baseline cardiometabolic disease (CMD) status (*n =* 40,080)

Characteristics	CMD-free (*n =* 34302)	Single CMD (*n =* 4809)	CMD Multimorbidity (*n =* 969)	*P*-value
Age, years	59.2 ± 10.1	67.4 ± 11.1	72.5 ± 9.3	<0.001
Sex				<0.001
Male	15489 (45.2)	2662 (55.4)	564 (58.2)	
Female	18813 (54.8)	2147 (44.6)	405 (41.8)	
Education				<0.001
<8 years	11628 (33.9)	2385 (49.6)	596 (61.5)	
≥8 years	22674 (66.1)	2424 (50.4)	373 (38.5)	
Marital status				<0.001
Married/cohabitating	25338 (73.9)	3168 (65.9)	595 (61.4)	
Single	8964 (26.1)	1641 (34.1)	374 (38.6)	
Zygosity				<0.001
Monozygotic	6778 (19.8)	1005 (20.9)	198 (20.4)	
Dizygotic	22937 (66.8)	3247 (67.5)	681 (70.3)	
Undetermined	4587 (13.4)	557 (11.6)	90 (9.3)	
BMI, kg/m^2^	24.9 ± 3.4	25.8 ± 3.8	26.2 ± 5.4	<0.001
Smoking				0.862
Non–smokers	17011 (49.6)	2396 (49.8)	488 (50.4)	
Current/former smokers	17291 (50.4)	2413 (50.2)	481 (49.6)	
Alcohol consumption				0.657
No/mild drinking	31998 (93.3)	4470 (93.0)	901 (93.0)	
Heavy drinking	2304 (6.7)	339 (7.0)	68 (7.0)	
Physical activity level				<0.001
Low	8057 (23.5)	1122 (23.3)	280 (28.9)	
High	26245 (76.5)	3687 (76.7)	689 (71.1)	
Hypertension	5385 (15.7)	689 (14.3)	316 (32.6)	<0.001

*Note:* Data are presented as mean ± standard deviation or number (proportion, %).

### Association between CMDs and depression

During the follow-up (median: 15.9 years, interquartile range: 14.4 to 17.3 years), 1361 (3.4%) participants developed depression. In Cox models, compared with CMD-free participants, those with any CMD (HR 1.55, 95% CI, 1.35–1.78), a single CMD (HR 1.52, 95% CI, 1.31–1.76), or cardiometabolic multimorbidity (HR 1.83, 95% CI, 1.29–2.58) had an increased risk of depression. Further, there was a significant dose-dependent relationship between number of co-morbid CMDs (i.e., 0, 1, ≥2 CMD) and depression risk, indicating that the risk of depression increased by 43% with each additional co-morbid CMD (HR = 1.43, 95% CI, 1.28–1.60). Regarding specific CMDs and CMD combinations, diabetes only, heart disease only, co-morbid diabetes/heart disease, and co-morbid heart disease/stroke were associated with an increased risk of depression (Table 2).

**Table 2.** Hazard ratios (HRs) and 95% confidence intervals (CIs) for the association between cardiometabolic disease (CMD) and depression risk

CMD	No. of subjects	Depression		
		No. of cases	Basic-adjusted HR (95% CI)^a^	Multi-adjusted HR (95% CI)^b^
CMD–free	34302	1095	Reference	Reference
Any CMD	5778	266	1.61 (1.40–1.85)	1.55 (1.35–1.78)
Single CMD	4809	232	1.53 (1.32–1.77)	1.52 (1.31–1.76)
Diabetes alone	1543	66	1.30 (1.01–1.67)	1.29 (1.01–1.66)
Heart disease alone	2685	143	1.78 (1.49–2.13)	1.71 (1.43–2.04)
Stroke alone	581	23	1.43 (0.95–2.17)	1.31 (0.87–1.99)
CMD multimorbidity	969	34	1.86 (1.32–2.63)	1.83 (1.29–2.58)
Diabetes + heart disease	484	18	1.82 (1.14–2.91)	1.75 (1.10–2.80)
Heart disease + stroke	294	11	2.62 (1.44–4.76)	2.44 (1.34–4.43)
Diabetes + stroke	104	3	1.39 (0.45–4.31)	1.17 (0.38–3.64)
Diabetes + heart disease + stroke	87	2	1.80 (0.45–7.20)	1.61 (0.40–6.44)
Hazard ratio of depression per each additional co–morbid CMD	–	–	1.44 (1.29–1.61)	1.43 (1.28–1.60)

a Models were adjusted for age, sex, and education.

b Models were adjusted for age, sex, education, marital status, body mass index, smoking status, alcohol consumption, physical activity level, and hypertension.

**Figure 2.**
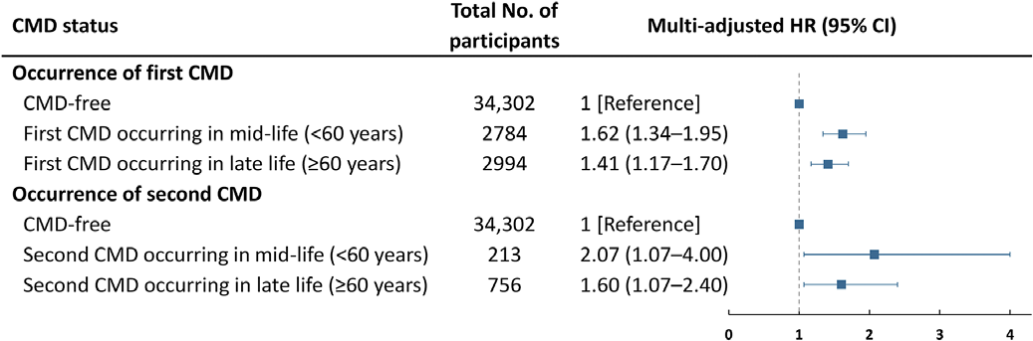
Risk of incident depression in relation to the age of cardiometabolic disease (CMD) occurrence. Models were adjusted for age, sex, education, marital status, body mass index, smoking status, alcohol consumption, physical activity level, and hypertension.

Furthermore, when accounting for the age of CMD occurrence, the risk of depression was higher if an individual’s first CMD occurred in mid-life (HR = 1.62, 95% CI, 1.34–1.95) as opposed to late life (HR = 1.41, 95% CI, 1.17–1.70). Additionally, the risk of depression appeared higher for those who subsequently developed a second CMD in mid-life (HR = 2.07, 95% CI, 1.07–4.00) compared with late life (HR = 1.60, 95% CI, 1.07–2.40) (Figure 2).

### Role of sex in the CMD-depression association

**Figure 3.**
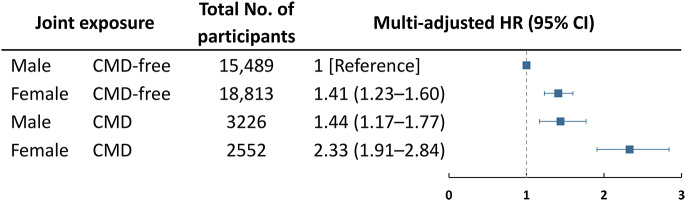
Risk of incident depression in relation to the joint effect of sex and cardiometabolic disease (CMD). Models were adjusted for age, education, marital status, body mass index, smoking status, alcohol consumption, physical activity level, and hypertension. Measures of additive interaction for depression: relative excess risk due to interaction 0.49, 95% CI, 0.02–0.96; attributable proportion due to interaction 0.21, 95% CI, 0.03–0.39.

After sex-stratification, the association between CMDs and depression remained significant among both men and women, and its magnitude tended to be stronger among women (Supplementary Table 2). However, we detected no significant multiplicative interaction between sex and CMDs on depression risk (*P* = 0.309). In joint effect analyses, the HRs of depression were 1.41 (95% CI ,1.23–1.60) for women without CMD, 1.44 (95% CI, 1.17–1.77) for men with CMDs, and 2.33 (95% CI, 1.91–2.84) for women with CMDs, compared with men without CMD (Figure 3). There was a significant additive interaction between sex and CMDs on depression (RERI = 0.49, 95% CI, 0.02–0.96; AP = 0.21, 95% CI, 0.03–0.39) (Supplementary Table 3).

### Roles of genetic background and early-life environment in the CMD-depression association

In matched co-twin analysis, the association between CMD and depression uncovered in the classical cohort study design (HR = 1.55, 95% CI, 1.35–1.78) remained present among DZ twin pairs (HR = 1.63, 95% CI, 1.02–2.59) but not MZ twin pairs (HR = 0.90, 95% CI, 0.32–2.51) (Table 3). Since the confounding influence of genetic factors could be controlled for among MZ twin pairs and early-life environmental factors could be controlled for among all twin pairs, this finding might suggest that genetic factors common to CMDs and depression may underlie the CMD-depression association.

**Table 3.** Hazard ratios (HRs) and 95% confidence intervals (CIs) for the association between cardiometabolic disease (CMD) and depression risk in matched co-twin analysis

Co-twin free of depression	Co-twin with depression			
	Dizygotic twins		Monozygotic twins	
	CMD	CMD-free	CMD	CMD-free
CMD	22	69	15	15
CMD–free	79	399	13	133
Basic–adjusted HR (95% CI)^a^	1.69 (1.01–2.68)		0.97 (0.24–2.64)	
Multi–adjusted HR (95% CI)^b^	1.63 (1.02–2.59)		0.90 (0.32–2.51)	

a Models adjusted for sex (if applicable) and education.

b Models adjusted for sex (if applicable), education, marital status, body mass index, smoking status, alcohol consumption, physical activity level, and hypertension.

### Sensitivity analysis

In sensitivity analyses, we obtained similar results when we repeated the analyses after excluding participants missing values for covariates (Supplementary Table 4) and excluding individuals diagnosed with depression within the first 2 years of follow-up (Supplementary Table 5). In addition, diabetes (HR 1.28, 95% CI, 1.03–2.60), heart disease (HR 1.75, 95% CI, 1.49–2.06), or stroke (HR 1.42, 95% CI, 1.03–1.95) was all individually associated with an increased risk of depression (Supplementary Table 6).

## Discussion

In this large-scale nationwide twin study, we found that 1) cardiometabolic multimorbidity, especially if developed in mid-life, was associated with an increased risk of depression; 2) the association between CMDs and depression risk was amplified among women; and 3) genetic and early-life environmental factors might account for the CMD-depression association.

Diabetes, heart disease, and stroke have all been related to an increased risk of depression [12–16]. However, the cumulative effect of co-morbid CMDs on depression risk has not been widely investigated. One cross-sectional study using data from the UK Biobank reported a monotonic increase in the likelihood of depression with a larger number of co-morbid CMDs (including diabetes, hypertension, stroke, and coronary artery disease) [17]. A previous cohort study of American veterans indicated that depression was more commonly detected among participants with co-morbid diabetes, heart disease, and/or hypertension than those with only one condition [25]. Another recent cohort study of middle-aged and older Chinese adults showed that cardiometabolic multimorbidity, compared to a single CMD, conferred a higher risk of depressive symptoms assessed with the Center for Epidemiological Studies Depression Scale [26]. In line with this literature, we found a dose-dependent relationship between number of co-morbid CMDs and depression risk in late life. Specifically, the risk of depression increased by 43% with each additional co-morbid CMD.

Our study took the further steps of exploring the effect of the timing of CMD occurrence across the lifespan on depression risk and the sex difference in the CMDs-depression association. Our results indicate that CMDs may have a stronger influence on depression risk if they occurred in mid-life as opposed to late life. This finding may support the “on-time, off-time” hypothesis that some risk factors such as CMD might have larger impact on depression if they occur at a more unexpected point across the lifespan [27]. That is to say, although CMD are more common in later life, the earlier CMDs set in, the more detrimental they may be for mental health.

In addition, we observed that the CMDs-depression relationship seemed to be strengthened among women. Although there was no multiplicative interaction between sex and CMDs on depression, we found that 21% of depression risk might be attributed to the additive interactions between female sex and CMDs, indicating that this joint effect was greater than the sum of the two individual effects. Women already have a higher risk of depression than men [28], and it is possible that this is exacerbated in the presence of CMDs. These findings have important public health implications; special monitoring and management of CMDs may not only benefit mental health in the general population, but may also be more effective when targeted at women and those who developed CMDs earlier in life.

Although it is widely accepted that genetic background and early-life environmental factors are implicated in the development of CMDs and depression, few studies have assessed the roles of these factors in the relationship between CMDs and depression [13, 14]. A study from the Danish Twin Registry reported that the increased risk of depression related to ischemic heart disease or stroke was independent of zygosity status and thus not explained by genetic factors [14]. On the other hand, another study composed of full siblings and maternal half-sibling pairs showed that genetic factors might contribute to the association between early-onset diabetes (diagnosed before age 45) and depression [13]. In our matched co-twin analysis, we found that the CMD-depression association originally observed in the classical cohort study design remained significant in DZ twin pairs but was attenuated and became non-significant in MZ twin pairs. This pattern of results suggests that the observed association between CMDs and depression might be in part influenced by genetic background. However, given the relatively small sample size of MZ twin pairs available in our study population and thereby lower statistical power, the CIs for the HR estimates in MZ and DZ twin pairs largely overlapped. This limits the interpretation and generalizability of our findings from the MZ analysis; additional studies are needed to clarify the role of genetic factors in the CMD-depression association.

Several explanations have been proposed for the association between cardiometabolic multimorbidity and depression. The “vascular depression” hypothesis posits that lesions related to vascular disease (including cardiovascular events and stroke) and vascular risk factors contribute to late-life depressive syndromes by disrupting prefrontal systems or their modulating pathways [29]. Specifically, the accumulation of vascular lesions may lead to impaired neural connectivity, cerebral hypoperfusion, and white matter abnormalities, thereby exerting adverse effects on brain function that influences the development of depression [30]. The neurochemical alterations related to stroke, such as changes in brain neurotransmitters and the downregulation of neurotrophic factors, can also result in depression [31]. Additionally, the neuroendocrine dysfunction of the hypothalamic–pituitary–adrenal axis and sympathetic nervous system might underpin the association between diabetes and depression [32, 33]. Furthermore, diabetes, heart disease, and stroke alike may bring about chronic stress and increased inflammation which are responsible for mental disorders including depression [30, 32–34]. Behaviorally, CMDs not only increase people’s psychological burden [35], but also share some lifestyle risk factors with depression, including smoking, obesity, sedentary lifestyle, physical inactivity, and poor sleep [10, 36, 37].

Strengths of this study include the use of longitudinal data from a large, nationally representative cohort of twin individuals. Additionally, the genetically informative twin study design allowed us to explore the roles of genetic and early-life environmental factors in the CMD-depression association. However, several limitations should be acknowledged. First, Swedish NPR only includes records from inpatient and outpatient clinics, so we could not capture diagnoses of CMDs or depression that occurred in the primary care setting. Additionally, some undiagnosed cases of CMDs and depression were likely missed. Second, the number of cases of incident depression among some CMD groups (such as stroke only and co-morbid diabetes/stroke) in the classical cohort study design and among MZ twin pairs in the matched co-twin analysis was small, and therefore statistical power was limited to detect a significant difference in depression risk. Third, although we considered many potential confounders, some factors (such as social activity engagement and sleep patterns) related to CMDs or depression [37, 38] could not be considered due to data unavailability.

In conclusion, our study provides evidence that the presence of CMDs is dose-dependently associated with an elevated risk of depression. Women and individuals who developed CMDs in mid-life have higher depression risk. Genetic background may underpin the association between CMDs and depression. Our study highlights active management of CMDs as a potential strategy for prevention of depression and underscores the need for mental health resources for individuals with CMDs, especially those with cardiometabolic multimorbidity.

## Supporting information

Yang et al. supplementary material Yang et al. supplementary material

## Data Availability

The data that support the findings of this study are available from the corresponding author upon reasonable request.

## Supplementary material

The supplementary material for this article can be found at http://doi.org/10.1192/j.eurpsy.2024.1775.
